# Non-destructive Measurement of Calcium and Potassium in Apple and Pear Using Handheld X-ray Fluorescence

**DOI:** 10.3389/fpls.2016.00442

**Published:** 2016-04-05

**Authors:** Lee A. Kalcsits

**Affiliations:** ^1^Tree Fruit Research and Extension Center, Washington State UniversityWenatchee, WA, USA; ^2^Department of Horticulture, Washington State UniversityPullman, WA, USA

**Keywords:** X-ray fluorescence, apple, pear, calcium, potassium, Non-destructive, semi-quantitative

## Abstract

Calcium and potassium are essential for cell signaling, ion homeostasis and cell wall strength in plants. Unlike nutrients such as nitrogen and potassium, calcium is immobile in plants. Localized calcium deficiencies result in agricultural losses; particularly for fleshy horticultural crops in which elemental imbalances in fruit contribute to the development of physiological disorders such as bitter pit in apple and cork spot in pear. Currently, elemental analysis of plant tissue is destructive, time consuming and costly. This is a limitation for nutrition studies related to calcium in plants. Handheld portable x-ray fluorescence (XRF) can be used to non-destructively measure elemental concentrations. The main objective was to test if handheld XRF can be used for semi-quantitative calcium and potassium analysis of in-tact apple and pear. Semi-quantitative measurements for individual fruit were compared to results obtained from traditional lab analysis. Here, we observed significant correlations between handheld XRF measurements of calcium and potassium and concentrations determined using MP-AES lab analysis. Pearson correlation coefficients ranged from 0.73 and 0.97. Furthermore, measuring apple and pear using handheld XRF identified spatial variability in calcium and potassium concentrations on the surface of individual fruit. This variability may contribute to the development of localized nutritional imbalances. This highlights the importance of understanding spatial and temporal variability in elemental concentrations in plant tissue. Handheld XRF is a relatively high-throughput approach for measuring calcium and potassium in plant tissue. It can be used in conjunction with traditional lab analysis to better understand spatial and temporal patterns in calcium and potassium uptake and distribution within an organ, plant or across the landscape.

## Introduction

Calcium and potassium are critical macronutrients for plants and essential for cell wall synthesis, signaling processes and cellular homeostasis (Marschner, 2011). In horticultural crops, localized calcium deficiencies are common and result in postharvest quality reduction and economic losses (White and Broadley, 2003). Low calcium in fruit tissue has been associated with disorders such as blossom end-rot in tomato (*Solanum lycopersicum* L.) (Ho and White, 2005) and pepper (*Capsicum annuum* L.) (Marcelis and Ho, 1999), hollow-heart in potato (*Solanum tuberosum* L.) (Palta, 2010), bitter pit in apple (*Malus domestica* Borkh.) (Ferguson and Watkins, 1992; de Freitas et al., 2010), and cork spot in pear (*Pyrus communis* L.) (Mason and Welsh, 1970; Raese and Drake, 2006), among others. These disorders limit the productivity of horticultural crops. Despite over 100 years of research toward addressing these issues, the physiological mechanisms underpinning the development of these disorders are still poorly understood (Ho and White, 2005; de Freitas et al., 2010; Saure, 2014). Advances in the rate of elemental measurements are required to increase the capacity to understand the complexity of localized nutritional imbalances in fleshy horticultural plants that contribute to calcium-related disorders.

The supply of calcium to a developing plant organ is dependent upon uptake from the soil and transfer via the xylem. Upon transfer from the xylem to cells surrounding the xylem, the mobility of an element is a function of the combined symplastic and apoplastic flow (Gilliham et al., 2011). Using ^45^Ca radioisotope, Shear and Faust (1970) showed that calcium mobility is limited in plant tissue once it is unloaded from the xylem and calcium accumulates in cells adjacent to xylem vessels. Recent advancements have increased the resolution at which we can measure these spatial differences in microstructure that can affect calcium mobility. Herremans et al. (2015) used x-ray tomography to map vascular connectivity networks in an apple fruit. These complex three dimensional images can bring to light potential calcium-limited regions that are the furthest distance from xylem vessels in the apple. These occur nearest the calyx end and immediately under the peel. Differences in the distribution of xylem vessels and functionality over time (Drazeta et al., 2004) can cause a large amount of spatial variability in plant tissue elemental concentrations. Indeed, this has been shown using traditional elemental analysis (Ferguson and Watkins, 1983). In apple fruit, calcium concentrations are higher in apple mesocarp and the concentration decreases with distance from the center of the fruit (Ferguson and Watkins, 1983). The apple peel has a high concentration relative to the flesh (Perring and Pearson, 1986). To properly assess the risk of the development of calcium-deficiency related disorders, the calcium-limited areas must be properly identified and measured.

At the cellular level, there is a better understanding of the contribution of calcium to cell wall strength of horticultural crops and its contribution to quality and resistance to postharvest disorders (de Freitas et al., 2010, 2012). Calcium binds with pectic acids to form calcium pectates which are present in both the cell wall and intercellular space (de Freitas et al., 2010). Approximately 65–70% of cellular calcium is concentrated in the cell wall (Demarty et al., 1984). If apoplastic calcium concentrations are too low, cell membrane leakage and damage can occur (Picchioni et al., 1998). Although the role of calcium in cell wall strength is relatively well constrained, there has been little progress in understanding the underlying physiological mechanisms contributing to abnormal cellular calcium partitioning that leads to calcium-related physiological disorders in horticultural crops. Cytoplasmic and vacuolar calcium concentration are normally much lower than the cell wall (Poovaiah, 1988; Hepler, 2005). However, during calcium signaling events, calcium concentrations can increase several fold (Hepler, 2005). The pools of calcium for these sudden shifts in calcium concentrations are either apoplastic calcium, calcium stored in the vacuole or a combination of these two pools. The vacuole is considered to be a largely unidirectional storage sink for calcium and potassium where large amounts of calcium and potassium can be stored but are rarely transferred to other parts of the cell. Vacuolar calcium concentrations can be high and are relatively unavailable for metabolic processes. This can create uncertainty when linking tissue elemental concentrations to localized deficiencies in the apoplastic space. Free apoplastic calcium has been suggested to contribute to the development of cell leakage and damage that precedes the development of symptoms such as blossom end-rot in tomato (de Freitas et al., 2012) and bitter pit in apple (Harker and Venis, 1991; Ho and White, 2005; de Freitas et al., 2010). Still, the overall calcium concentration in plant tissue and its ratio to other elements, such as potassium, magnesium and nitrogen, have been implicated as a predictor of calcium-deficiency related physiological disorders that occur in horticultural crops (White and Broadley, 2003).

The most common method of analyzing calcium and potassium concentrations in plant tissue is through laboratory analysis that often includes time-consuming sample drying, homogenization and acid digestion. Cost, time, strong interactions between calcium nutrition and environmental conditions (Saure, 2001; de Freitas et al., 2011), and agronomic management (Wünsche and Ferguson, 2005) limit the ability to expand upon the extensive amount of research in this area. Furthermore, sample pooling within replicates limits the amount of information that can be acquired, particularly when elemental concentrations can vary throughout a fruit (Ferguson and Watkins, 1983; Perring and Pearson, 1986), plant or field. Recently, non-destructive analytical approaches have become available that have the potential to be field portable to measure calcium and potassium in fleshy fruit. Handheld x-ray fluorescence (XRF) has been more commonly used for metal identification and heavier elements (Bennett and Oliver, 1992). However, with recent technological advances such as vacuum attachments and more sensitive detection diodes, this technology has the potential to be used for the non-destructive detection of lighter elements in plants (McLaren et al., 2012; Reidinger et al., 2012) including calcium and potassium.

In recent years, the usefulness of using handheld XRF has been demonstrated in manufacturing (Marguí et al., 2012), archeology (Nazaroff et al., 2010) and on a limited destructive basis, soil and plant sciences (Zhu and Weindorf, 2009; McLaren et al., 2012; Reidinger et al., 2012). Zhu and Weindorf (2009) used a field portable handheld portable x-ray fluorometer (PXRF) to measure calcium in soils. In plants, Reidinger et al. (2012) used a handheld PXRF to quantify phosphorus and silica in homogenized and pelletized plant tissue. McLaren et al. (2012) reported significant correlations between traditional digested plant tissue measurements and those made using a handheld PXRF for analysis of ground, pelletized plant tissue samples across a range of elements including calcium and potassium. For elements such as potassium and calcium that are present in high concentrations in plant tissue, a handheld PXRF could be used as a non-destructive instrument to semi-quantitatively measure calcium and potassium in plants. Here, the main objective was to determine if handheld XRF can be used to non-destructively assay calcium and potassium concentrations in apple and pear *in-situ*. Field portable, non-destructive semi-quantitative analysis would allow for increased replication and more complex sampling to better examine the spatial distribution of calcium and potassium at the organ, plant or field level. A relatively large increase in the capacity to measure calcium and potassium would be valuable to better understand calcium and potassium uptake and distribution in plants and the influence of environment and agronomic practices on these patterns.

## Materials and Methods

### Plant Material

Fruit material was chosen for analysis to reflect a range in calcium and potassium concentrations. ‘Honeycrisp’ apples were obtained from two growing regions in Washington State and were harvested in 2014. Fifty four apples were selected for analysis from fields that were known to reflect historical differences in calcium concentration and bitter pit incidence. The fruit was stored at 2°C until analysis with the handheld PXRF. Similar to apples, 80 pears (‘Starkrimson,’ ‘Bartlett’) were selected that contain a range in fruit calcium and potassium concentrations from fields near Wenatchee, WA, USA. All fruit was uniform in size and disorder-free. Fruit was triple-washed with ultrapure water to remove any residual calcium on the peel of the fruit prior to analysis.

### Handheld X-ray Fluorescence

**FIGURE 1:**
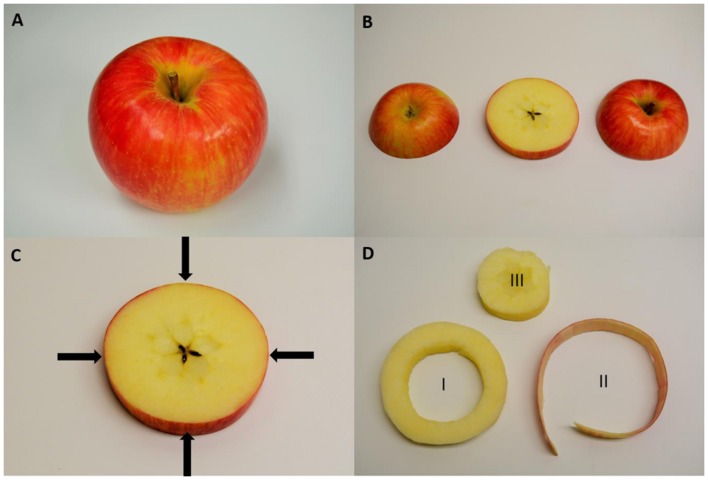
**Sampling protocol of a ‘Honeycrisp’ apple for handheld x-ray fluorescence (XRF) and destructive lab sampling. (A)** Whole apple where four measurements were made with the handheld XRF along the equator of the fruit. **(B)** To compare handheld XRF, an equatorial slice was removed from the apple. **(C)** A representation of the sampling locations for handheld XRF analysis. **(D)** Destructive sampling for elemental analysis of (I) the outer cortex of the apple used for analysis of homogenized pellets using the handheld XRF and then digesting for lab elemental analysis, (II) apple peel to compare with handheld XRF measurements made along the equatorial region of the fruit while it was whole, and (III) the core of the apple which was discarded.

Apples and pears were analyzed using a Bruker AXS Tracer 3-V Portable handheld x-ray fluorometer (PXRF) analyzer (Bruker Elemental, Kennewick, WA, USA), equipped with a rhodium tube from which X-rays are emitted, and a peltier-cooled, silicon PIN diode detector, operating at 15 kV and 25 μA from an external power source for 15 s using no filter under a vacuum at <10 torr. This instrument operates at low power and therefore is portable and handheld. The Bruker instrument produces an X-ray beam at a 45° angle from the center of the analyzer’s tip (**Figure 1**; adapted from Nazaroff et al., 2010). In order to make sure that analysis of each sample included the bulk of the X-rays produced, each sample was placed to cover the 45° angle beam path. The beam covered an area with a diameter of 8 mm. Samples were positioned with as much contact as possible with the instrument’s surface. This ensures that the greatest amount of X-rays possible would bombard the sample, which would optimize the count rate and mitigate the effects of irregular sample surface structure on X-ray scatter. The estimated penetration depth for the counts measured for calcium and potassium was approximately 1 mm which would include the peel and immediate outer cortex of the fruit. X-ray counts were processed using the S1PXRF spectra program developed by Bruker and used as a semi-quantitative approach for measuring calcium and potassium. Each fruit was measured at four different spots along the equator.

### Destructive Sample Processing and MP-AES Analysis

Once apples were measured using the handheld PXRF, a 1 cm wide slice was taken at the equator of each fruit (**Figure 1**) and a 1 mm band, including the fruit peel, was removed. A 1 cm thick sample of the cortex was also removed from the equatorial slice to use for comparing pelletized flesh tissue with traditional lab analysis. Each sample was oven-dried at 60 C for 3 days and ground to micron size using a VWR Homogenizer (VWR, Radnor, PA, USA). Two hundred miligram of sample was digested with 6 mL of HNO_3_ and the digest was filtered with a 0.45 μM PTFE filter. Filtered digests were diluted 100x and analyzed at Washington State University soil chemistry service lab using a MP-AES and run in combination with calcium and potassium ICP standards for validation. The concentration of calcium and potassium standards were chosen to bracket the approximate concentrations of the digests for potassium and calcium.

### Handheld X-ray Fluorescence Analysis of Pelletized Apple Tissue

Using the homogenized cortex samples, 1 g of plant tissue was pressed in a Parr pellet press (Parr Instrument Company, Moline, IL, USA). The pellet was analyzed using the handheld PXRF using the same protocols as stated above on the upper and lower side of the pellet. The average value was used for each sample. This was also done for another subset of Honeycrisp apple samples (*N*=54) for a total sample number of 128 to compare non-destructive measurements of calcium and potassium using the handheld PXRF with traditional lab analysis.

### XRF Analysis of Agar Disks with Known Calcium and Potassium Concentrations

Here, to test the use of the handheld PXRF in detecting calcium and potassium in a carbon-water matrix, similar to plants, agar disks were used. To test the influence of agar concentration on measurements, a subset of disks were prepared at 100, 1000, and 2500 mg L^-1^ Ca and K using 1, 2, and 3% agar solutions. There were no effects of agar concentration on the detection of calcium or potassium in each disk. Then, eight different sets (*n* = 3) of 3% agar gel disks were prepared with a known range in combined calcium and potassium concentrations (0, 10, 25, 50, 100, 500, 1250, 2500, and 5000 mg L^-1^ for Ca and correspondingly, 0, 100, 250, 500, 1000, 2500, 6250, 12,500, and 25,000 mg L^-1^ for K) using calcium nitrate and potassium nitrate as sources for calcium and potassium, respectively. These concentrations were chosen to reflect the known natural variation in calcium and potassium in fruit. Agar disks were prepared by boiling in a microwave three times in 15 s increments until the agar was completely dissolved. The agar was then poured into petri-dishes and left to solidify. Disks were then analyzed using the PXRF according to the protocol used previously for fruit tissue. X-ray counts were processed using the S1PXRF Spectra program developed by Bruker and used as a semi-quantitative relative comparison of calcium and potassium.

### Mapping of Calcium and Potassium Concentrations for Healthy and Bitter Pit Affected Fruit and Apple Leaves

To estimate the calcium and potassium distribution in apple fruit and leaves, a grid was created on the surface of two fruit; one showing symptoms of bitter pit and one that was healthy. Sampling locations on the fruit were assigned a longitude and latitude on the surface of the fruit for a total of 180 analysis locations for each fruit (20° longitude and 18° latitude from one measurement point to another). Using an embedded smoothing algorithm in Origin Pro 15 (Origin Lab, Northampton, MA, USA), data was smoothed and then overlaid on a three dimensional sphere, oriented to where the highest point represents the stem end and the lowest point represents the calyx end.

### Data Analysis

X-ray counts were processed using the Bruker handheld PXRF count processing program, ARTAX (Bruker Elemental, Kennewick, WA, USA). Calcium and potassium counts were delineated using a Bayesian deconvolution for each element and then normalized to the rhodium peak. In many cases, normalization does not have an appreciable impact but can be effective at reducing some of the inherent variability present in the x-ray counts. These standardized counts act as semi-quantitative indicators of the presence of potassium or calcium in the instrument. Because the measurements are non-destructive, it is not possible to use standard references to calibrate the instrument for quantitative use. Calibrations that can be developed will require spiking or identifying in-tact samples with high differences in calcium that bracket biologically realistic calcium and potassium concentrations in leaf and fruit tissue. The mean rhodium-normalized count rate for calcium and potassium in each fruit was calculated from the four equatorial measurements to semi-quantitatively estimate the calcium and potassium in the peel and immediate outer flesh of the fruit. The mean non-destructive measurements was then compared to the digested MP-OES lab analysis using Origin Pro 15 (Origin Lab, Northampton, MA, USA).

## Results And Discussion

### Normalized Counts from Handheld XRF Analysis of Controlled Calcium and Potassium Spiked Agar-Gel Are Strongly Associated with Known Calcium and Potassium Concentrations in the Matrix

**FIGURE 2:**
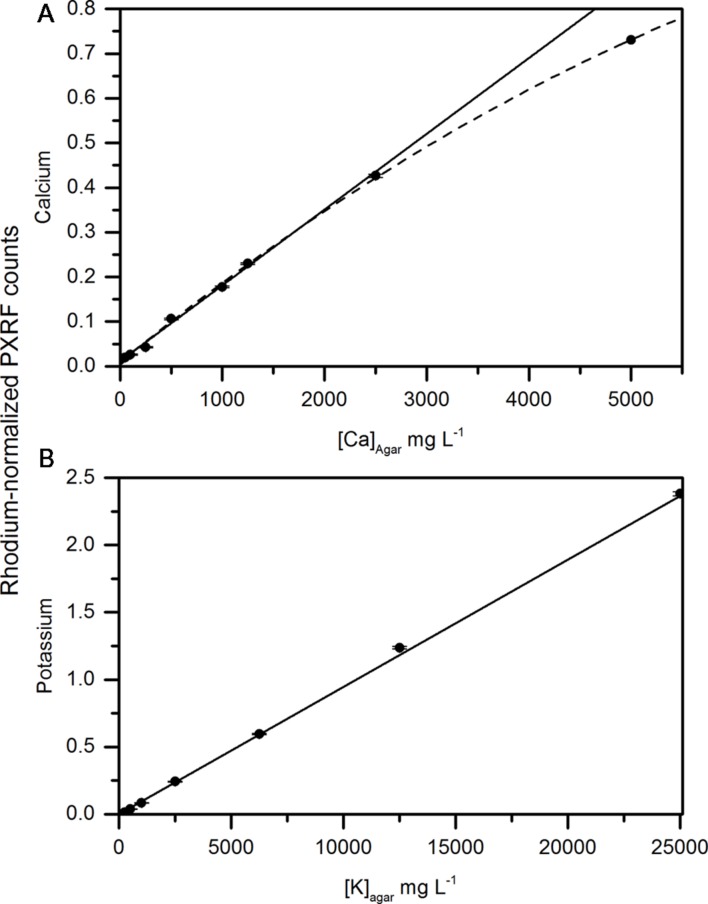
**Correlation between concentrations of **(A)** calcium and **(B)** potassium in an agar matrix measured using the handheld x-ray fluorometer.** The solid line represents the linear fit of calcium and potassium concentrations in the agar up to 2500 and 12,500 mg L^-1^, respectively. The dashed line represents the non-linear fit of agar disks containing calcium up to 5000 mg L^-1^.

Early assessment of handheld XRF for non-destructive analysis was focused on whether carbon- and water-containing matrices affected the detection of calcium and potassium in a solid matrix. Reidinger et al. (2012) performed a similar analysis using dry methyl cellulose pellets spiked with either silicon or phosphorus. Here, we wanted to measure calcium and potassium in the presence of water. So instead, we used an agar gel base for the spiked samples. There was a strong relationship between the concentrations of calcium and potassium and the rhodium-normalized counts measured using the PXRF (**Figures 2A,B**). Potassium concentrations were distinctly linear (**Figure 2B**) (*r* = 0.997, *P* < 0.001). The relationship between delineated counts using the PXRF and known concentrations of calcium in the agar disks was also linear at low concentrations ([Ca]<2500 mg L^-1^) (**Figure 2A**). At high potassium and calcium concentrations ([Ca] = 5000 mg L^-1^), the relationship between calcium counts using the PXRF and known calcium concentrations became non-linear. This may be a result of the secondary potassium peak interfering with the primary peak of calcium at correspondingly high concentrations of potassium (>12,500 mg L^-1^). The relationship between calcium and the normalized counts becomes linear in the absence of high concentrations of potassium indicating that there is an interference from potassium on the analysis of calcium only when potassium is present in high concentrations. However, potassium concentrations in plant tissue rarely exceeds 10000 mg kg-1 DW concentrations (Estimated from Ferguson and Watkins, 1983) and therefore, we would not expect significant interferences from potassium on calcium measurements made on fruit *in situ*. If concentrations are higher than 10000 mg Kg^-1^ DW, there is a risk of interferences. The bayesien deconvolution analysis embedded in the software minimizes these interferences. While destructive sample homogenization of dried samples or NIST standards used in previous studies is more reproducible (Reidinger et al., 2012), recreation of water-cellulose matrix spiked with known standards provided validation of non-destructive, whole tissue elemental analysis using a handheld PXRF.

### Semi-quantitative, Non-destructive Measurements Using a Handheld PXRF Are Correlated with MP-AES Analysis of Calcium and Potassium

**FIGURE 3:**
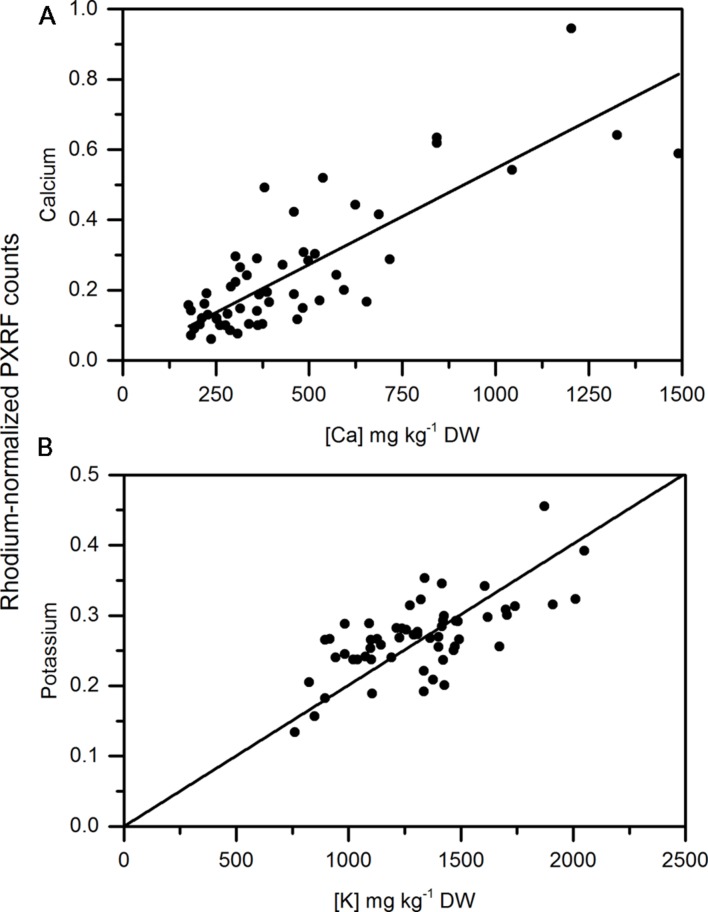
**Correlation of non-destructive, semi-quantitative mean **(A)** calcium and **(B)** potassium content [mg kg^-1^ dry weight (DW)] compared to corresponding MP-AES analysis of calcium in apple peel (*N* = 54)**.

**FIGURE 4:**
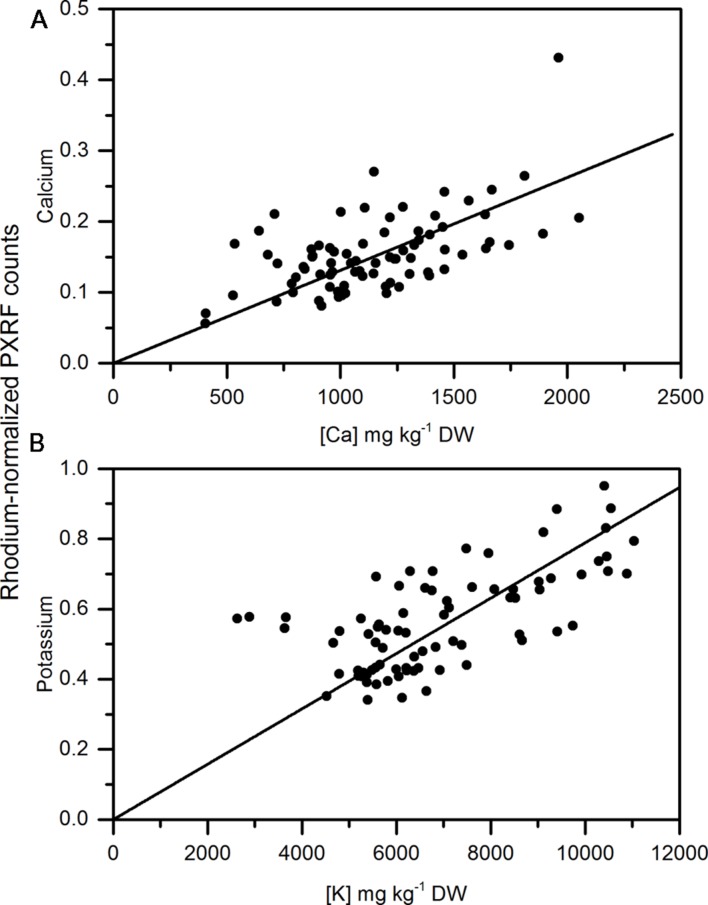
**Correlation of non-destructive, semi-quantitative mean **(A)** calcium and **(B)** potassium content compared to corresponding MP-AES analysis of calcium in pear peel (*N* = 80)**.

Calcium concentrations in apple peel tissue ranged by a factor of 10 from approximately 150 mg kg-1 DW to 1500 mg kg-1 dw. Semi-quantitiative measurements ranged by equivalent orders of magnitude in the apple samples. Semi-quantitative measurements of calcium using the handheld PXRF measured on four spots around the equator of the fruit were significantly correlated with the calcium concentrations in the entire peel (See **Figure 1** for a description of sampling) surrounding the equator of the apple (**Figure 3A**) (*r* = 0.941, *P* < 0.001). Similarly, when potassium concentrations were measured using MP-OES in apple tissue measured with the PXRF, there was a significant correlation (**Figure 3B**) (*r* = 0.986, *P* < 0.001). High potassium to calcium ratios in fruit has been previously used as an indicator of bitter pit susceptibility (Ferguson and Watkins, 1983; Perring and Pearson, 1986). In pears, calcium and potassium concentrations in individual fruit were strongly correlated with non-destructive XRF measurements. The Pearson correlation coefficients were 0.958 and 0.977 for XRF measurements of calcium and potassium compared to digestion analysis using MP-AES (**Figures 4A,B**, respectively). However, the slope of the regression lines for the correlation between lab and XRF analysis was less for calcium and potassium in pears than the slope of the regression line for calcium and potassium in apples.

**FIGURE 5:**
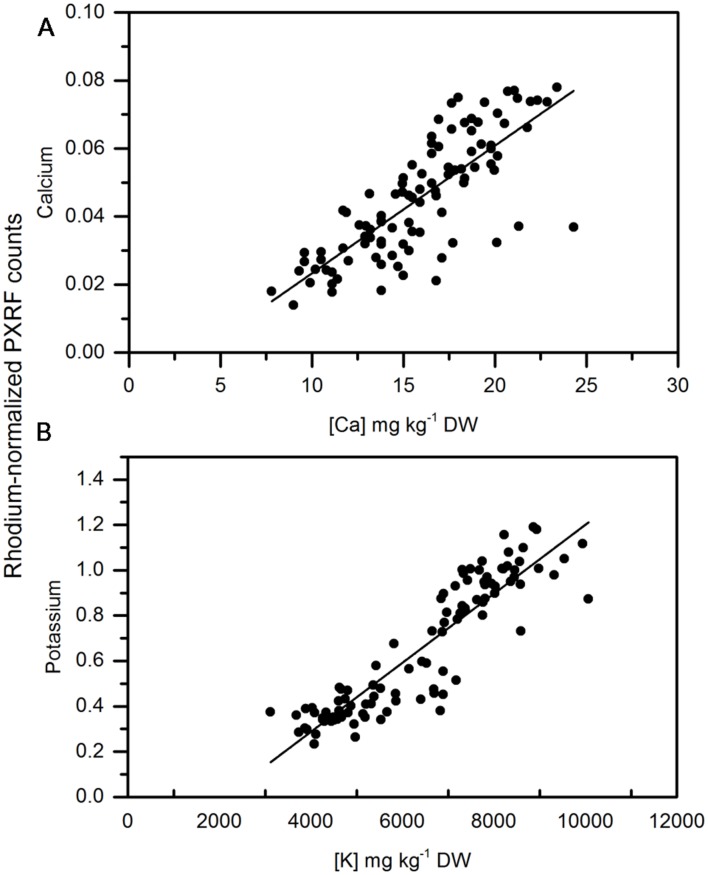
**Correlation of non-destructive, semi-quantitative mean **(A)** calcium and **(B)** Potassium content of pelletized, ground apple cortex tissue compared to corresponding MP-AES analysis of calcium. (*N* = 104)**.

As a more conventional comparison of apple and pear tissue among measurements and similar to Reidinger et al. (2012), homogenized tissue of apple from two different regions in Washington State were analyzed using the handheld PXRF. Cortex samples had lower amounts of calcium than the peel for apple and were less variable than when analyzing the peel. In general, the region of the apple with the lowest concentrations of calcium is in the outer cortex (Wünsche and Ferguson, 2005) and is similar to what was measured here using handheld XRF analysis of pelletized outer cortex tissue. Similar to the non-destructive measurements made on the peel, there was a significant relationship between the pelleted homogenized samples and MP-OES lab analysis (**Figures 5A,B**). Pearson correlation coefficients were 0.787 and 0.89 for calcium and potassium, respectively. Reidinger et al. (2012) reported high precision in analyzing phosphorus and silicon using this approach. McLaren et al. (2012) also reported high correlations between homogenized samples and lab analysis of leaves from four different species of plants. However, homogenization still represents a destructive approach to sampling. In specific cases, such as calcium, which can be locally deficient within the plant, non-destructive analysis is more appropriate since variation between tissues and organs (in the case of non-destructive handheld XRF) may be more important than a pooled value from completely homogenized tissue. However, for other elements and depending on the target location, homogenization and pelleting a sample may be more appropriate.

Calibration development for quantitative analysis would represent an advancement in the capability to use handheld XRF for non-destructive analysis. However, heterogeneity in cell type thickness and density among different cell types (i.e., epidermis versus cortex in an apple fruit) can lead to differences in x-ray penetration depth. Apple peel has been shown to vary among apple cultivars (Homutová and Blažek, 2006) and could impact XRF analysis. If peel thickness varies between species and/or genotypes, this will need to be investigated in more detail to understand how this contributes to elemental detection using XRF. Using epidermis and cortex as an example; if epidermal thickness was different among varieties of apple, the proportion of epidermis and cortex analyzed non-destructively using the handheld PXRF would also be different. Since the elemental concentrations are different between the epidermis and the cortex, differences in the proportions of each tissue analyzed using XRF should produce different results. Therefore a calibration developed for one variety would likely be different for another. Calibration development will likely require species and possible even cultivar-specific calibrations for the instrument. Semi-quantitative analysis is still always a possibility but comparisons would have to be among biologically similar samples for valid relative comparisons.

### Calcium and Potassium Are Unevenly Distributed within an Apple Fruit

**FIGURE 6:**
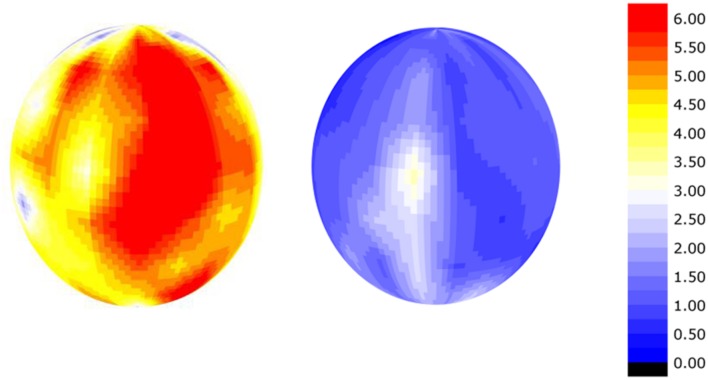
**Smoothed semi-quantitative potassium:calcium ratio on the surface of a apple affected by bitter-pit **(left)** and a healthy apple **(right)** calculated as the rhodium-normalized PXRF counts for potassium divided by the rhodium-normalized PXRF counts for calcium**.

There is a high amount of variation in calcium and potassium in the peel of apple fruit that was measured using handheld XRF. **Figure 6** shows the potassium to calcium ratio on the surface of a Honeycrisp apple affected by bitter pit compared to a healthy apple. The potassium to calcium ratios were almost twice as high in the bitter pit affected apple and the variation between different regions of the apple was also markedly different. The differences in these ratios were driven by both a higher presence of potassium and a lower presence of calcium. Calcium and potassium were as much as an order of magnitude different in one location on the fruit compared to another. There are several factors that can affect the elemental distribution within fruit. Lewis and Martin (1973) and Perring and Pearson (1986) demonstrated that calcium is lower on the calyx end of the fruit than the stem end. Furthermore, Xiaoyan et al. (2010) reported that calcium was greater on the sun-exposed portion of the apple and was again, higher on the stem-end than the calyx end of the fruit. Differences not only exist on the peel of an apple but also within the fruit. Ferguson and Watkins (1983) reported that flesh calcium concentrations decrease with increasing distance from the core. This may be, in part, related to the mode of distribution and proximity to xylem vessels in the fruit (de Freitas et al., 2012). Montanaro et al. (2014) demonstrated that xylem functionality in kiwi fruit contributes to fruit calcium uptake. Miqueloto et al. (2014) also reported the association between xylem functionality in apple and fruit elemental concentration and an association with bitter pit. Once calcium exits the xylem, it moves more slowly within the fruit. Because the mobility of calcium is limited, concentrations are thought to decrease with increasing distance from the cortex and distance from xylem vessels. Therefore, pockets on the outer cortex between xylem vessels should be the most susceptible to calcium-deficiency related disorders. With sufficient replication, the use of the handheld PXRF enables the study of the distribution of calcium and potassium throughout space within a fruit to identify these locally deficient regions of the fruit that may be the most susceptible to the development of physiological disorders related to calcium deficiencies.

## Conclusion

Handheld XRF has potential to be used as a semi-quantitative instrument that provides information to make relative comparisons on calcium and potassium concentrations amongst treatments with biologically similar samples. However, as a quantitative measure, there is still a need for the development of species-specific, or even cultivar-specific, calibrations. Here, we show the use of handheld XRF for semi-quantitative, non-destructive measurements of calcium and potassium. It provides opportunities to address different biological questions related to nutrient uptake and mobility that are not possible using traditional lab analysis or destructive homogenization. Using this approach, repeated measures are possible on the same biological sample through time. It also permits greater replication, reduced sampling time and more complex data sets that are often not feasible using traditional lab elemental analysis. Potential applications include improved precision in estimating changes in elemental concentration over time in plant tissue, analysis of the elemental distribution within an organ, within a tree or within a field when compared with traditional lab analysis. Handheld XRF is a viable alternative to compliment traditional lab elemental analysis which can improve the understanding of calcium and potassium dynamics in plants and make *in situ* non-destructive elemental measurements in the field.

## Author Contributions

LK participated in experimental design, analysis, implementation, data analysis, writing, and editing of the manuscript.

## Conflict of Interest Statement

The authors declare that the research was conducted in the absence of any commercial or financial relationships that could be construed as a potential conflict of interest.
